# Effects of salubrinal on development of osteoclasts and osteoblasts from bone marrow-derived cells

**DOI:** 10.1186/1471-2474-14-197

**Published:** 2013-07-01

**Authors:** Hiroki Yokota, Kazunori Hamamura, Andy Chen, Todd R Dodge, Nancy Tanjung, Aysan Abedinpoor, Ping Zhang

**Affiliations:** 1 Department of Biomedical Engineering, Indiana University-Purdue University Indianapolis, 723 West Michigan Street, SL220, Indianapolis, IN, 46202, USA; 2 Department of Anatomy and Cell Biology, Indiana University School of Medicine, Indianapolis, IN, 46202, USA; 3 School of Basic Medical Sciences, Tianjin Medical University, Tanjin, 300070, People’s Republic of China

**Keywords:** Osteoporosis, RANKL, Salubrinal, Osteoclasts, Osteoblasts

## Abstract

**Background:**

Osteoporosis is a skeletal disease leading to an increased risk of bone fracture. Using a mouse osteoporosis model induced by administration of a receptor activator of nuclear factor kappa-B ligand (RANKL), salubrinal was recently reported as a potential therapeutic agent. To evaluate the role of salubrinal in cellular fates as well as migratory and adhesive functions of osteoclast/osteoblast precursors, we examined the development of primary bone marrow-derived cells in the presence and absence of salubrinal. We addressed a question: are salubrinal’s actions more potent to the cells isolated from the osteoporotic mice than those isolated from the control mice?

**Methods:**

Using the RANKL-injected and control mice, bone marrow-derived cells were harvested. Osteoclastogenesis was induced by macrophage-colony stimulating factor and RANKL, while osteoblastogenesis was driven by dexamethasone, ascorbic acid, and β-glycerophosphate.

**Results:**

The results revealed that salubrinal suppressed the numbers of colony forming-unit (CFU)-granulocyte/macrophages and CFU-macrophages, as well as formation of mature osteoclasts in a dosage-dependent manner. Salubrinal also suppressed migration and adhesion of pre-osteoclasts and increased the number of CFU-osteoblasts. Salubrinal was more effective in exerting its effects in the cells isolated from the RANKL-injected mice than the control. Consistent with cellular fates and functions, salubrinal reduced the expression of nuclear factor of activated T cells c1 (NFATc1) as well as tartrate-resistant acid phosphatase.

**Conclusions:**

The results support the notion that salubrinal exhibits significant inhibition of osteoclastogenesis as well as stimulation of osteoblastogenesis in bone marrow-derived cells, and its efficacy is enhanced in the cells harvested from the osteoporotic bone samples.

## Background

Osteoporosis is a common skeletal disease of bone loss, which leads to an increased risk of bone fractures, morbidity, mortality, and an economic burden to society [1-3]. In many cases it is a physiological consequence of the aging process [3,4], and in postmenopausal women it is induced by a decrease in the production of estrogen, a hormone known to maintain the appropriate ratio of bone-forming osteoblasts to bone-resorbing osteoclasts [5]. During the past 20 years, many therapeutic drugs have been developed to prevent osteoporotic bone loss. Bisphosphonates are the most widely prescribed medications to treat postmenopausal osteoporosis, but they may be associated with an increased risk of osteonecrosis of the jawbone and atypical femur fracture [6]. Other treatments include administration of estrogen and estrogen analogs, as well as parathyroid hormone. However, increased risks of breast cancers and blood clots have been reported as side effects of these treatments [7-9]. The aim of this study is to evaluate a therapeutic role of a chemical agent, salubrinal, in potential treatment of osteoporosis.

Salubrinal is a small chemical agent (480 Da, C_21_H_17_Cl_3_N_4_OS) known to block de-phosphorylation of eukaryotic translation initiation factor 2 alpha (eIF2α) [10]. Salubrinal is also reported to attenuate molecular signaling mediated by nuclear factor kappa B (NFκB) [11]. The elevated phosphorylation level of eIF2α upregulates activating transcription factor 4 (ATF4), one of the key transcription factors in bone formation [12]. Salubrinal is shown to enhance healing of bone wounds and promotes differentiation of osteoblasts [13]. Little is known, however, about its effects on bone resorption, in particular developmental regulation of bone marrow-derived cells. Bone marrow-derived cells contain mesenchymal stem cells (MSCs) and hematopoietic stem cells that give rise to osteoblasts and osteoclasts, respectively [14]. The primary focus of this study is the potential role of salubrinal in the development of bone marrow-derived cells towards mature osteoclasts, as well as its role in development of mesenchymal stem cells and osteoblasts.

Experimental animal models are useful to evaluate therapeutic efficacy of chemical agents. Available osteoporosis models include ovariectomy (OVX) [15,16], tail suspension [17,18], denervation [19,20], a low-calcium diet [21,22], and administration of receptor activator of nuclear factor kappa-B ligand (RANKL) [23-25]. Any animal model may have its advantage and disadvantage. For instance, OVX-induced osteoporosis, which is currently considered as the gold standard for the evaluation of pharmaceuticals for postmenopausal osteoporosis, not only reduces the level of estrogen but also generates surgery-induced injury together with an increase in osteoblast activity. Furthermore, surgical induction of OVX requires consistency in the surgical procedure as well as a minimum of 4 weeks. The tail suspension model not only increases bone resorption but also reduces osteoblast differentiation. In the denervation model, surgery-induced injury is involved. In this study, we evaluated *in vivo* effects of salubrinal using the OVX mice and *in vitro* effects of salubrinal using bone marrow-derived cells isolated from the RANKL-injected mice.

In the RANKL administration model, RANKL is subcutaneously injected for as a short period as 3 days [26]. RANKL is a cytokine belonging to the tumor necrosis factor family. In the immune system, it is involved in dendritic cell maturation, while in the skeletal system it is a ligand for osteoprotegerin (OPG) and functions as a key regulator for osteoclast differentiation and activation [27,28]. RANKL deletion in mice leads to osteopetrosis and a decrease of osteoclasts, while RANKL overproduction is linked to a variety of degenerative bone diseases including osteoporosis and rheumatoid arthritis [29,30].

Focusing on the development of bone marrow-derived cells in the presence and absence of salubrinal, we addressed a pair of questions: Does administration of salubrinal modulate cellular fates and functions of bone marrow-derived cells in favor of prevention of bone loss? If so, are salubrinal’s actions more potent to the cells isolated from the osteoporotic RANKL-injected mice than those isolated from the control mice? Because of the anticipated role of salubrinal that is potentially opposite to that of RANKL, we hypothesized that salubrinal is more effective in inhibiting development of osteoclasts and stimulating development of osteoblasts in the cells isolated from the RANKL-injected mice than those from the control mice. To test the hypothesis, we employed assays such as colony-forming unit - granulocyte/macrophages (CFU-GM), colony-forming unit - macrophages (CFU-M), and formation of multi-nucleated osteoclasts in an osteoclast differentiation medium, as well as assays for migration and adhesion of pre-osteoclasts. We also conducted assays for examining colony-forming unit – osteoblasts (CFU-OBL) in an osteoblast differentiation medium. To evaluate salubrinal’s effects on expression of nuclear factor of activated T cells c1 (NFATc1), a master transcription factor for osteoclastogenesis, we conducted real-time PCR and Western blot analysis.

## Methods

### Animals and materials preparation

C57BL/6 female mice (7 weeks of age) were used. Each cage housed four to five mice at the Indiana University Animal Care Facility. They were fed with mouse chow and water *ad libitum*. Experimental procedures were approved by the Indiana University Animal Care and Use Committee and were in compliance with the Guiding Principles in the Care and Use of Animals endorsed by the American Physiological Society. Cytokines were purchased from PeproTech (Rocky Hills, NC, USA) and other chemicals from Sigma (St. Louis, MO, USA) unless otherwise stated. Salubrinal (R&D Systems, Minneapolis, MN, USA) was administered at 1 mg/kg to mice, and at 0.5 to 5 μM to cultured cells for the duration of each experiment.

### Ovariectomy

The animal was anesthetized with 1.5% isoflurane at a flow rate of 0.5 to 1.0 L/min. After removing the hair, the skin at the operative sites was cleaned using 70% alcohol and 10% providoneiodine solution. An incision (~20 mm) was made at the midline dorsal skin, and the peritoneal cavity was incised to access the ovaries. After removing the ovaries, the wound was closed by suturing. In 4 weeks after surgery, subcutaneous injection of salubrinal was conducted daily at a dose of 1 mg/kg body weight for 4 weeks. The control OVX mice received an equal volume of vehicle.

### RANKL administration for the bone loss model

Soluble recombinant murine RANKL (sRANKL; PeproTech) was injected subcutaneously using a 1 mg/kg dosage in 100 μl PBS at 24 h intervals for 3 days [26]. The same volume of PBS was injected into vehicle control mice. At 90 min after the final injection, the mice were euthanized. Iliac bones, femora, and tibiae were harvested, and bone marrow-derived cells were collected.

### Determination of bone mineral density (BMD) and bone mineral content (BMC)

The BMD (g/cm^2^) and BMC (g) of an entire humerus and ulna were determined using peripheral dual energy X-ray absorptiometry (DXA; PIXImus II, Lunar Corp., Madison, WI, USA) and its software (version 1.47).

### Colony-forming unit-granulocyte-macrophages (CFU-GM) assay

As previously described, a colony-forming unit-granulocyte-macrophage (CFU-GM) assay was conducted [31-33]. Approximately 2.5x10^4^ bone marrow-derived cells were prepared from the vehicle control and RANKL-treated mice and seeded onto a 35-mm gridded dish composed of methylcellulose supplemented with 30 ng/ml murine macrophage-colony stimulating factor (M-CSF), and 20 ng/ml RANKL. Three dosages of salubrinal (1, 2, and 5 μM) were administered, and cells were cultured at 37°C in a 5% CO_2_ incubator for 7 days.

### Colony-forming unit-macrophage/mononuclear (CFU-M) assay

Using bone marrow mononuclear cells (BMMNCs), a colony-forming unit-macrophage/mononuclear (CFU-M) assay was conducted, as described previously [34-37]. From the vehicle control and RANKL administration mice, approximately 2.5x10^4^ bone marrow-derived cells were prepared. Cells were seeded onto a 35-mm gridded dish, which was composed of methylcellulose supplemented with 30 ng/ml M-CSF and 20 ng/ml RANKL. Three dosages of salubrinal (1, 2, and 5 μM) were administered, and cells were cultured at 37°C in a 5% CO_2_ incubator for 7 days.

### Isolation of bone marrow-derived cells for osteoclast development

Bone marrow-derived cells were collected by flushing the iliac, femur and tibia with Iscove’s MEM (Gibco-Invitrogen, Carlsbad, CA, USA) containing 2% fetal bovine serum using a 23-gauge needle, as described previously [34,38]. Low-density gradient centrifugation was used to separate the cells, which were then cultured in α-MEM supplemented with 10% FBS, 30 ng/ml M-CSF, and 20 ng/ml murine receptor activator of nuclear factor kappa-B ligand (RANKL). Culture medium was replaced by α-MEM supplemented with 10% FBS, 30 ng/ml M-CSF, and 60 ng/ml RANKL on the third day, and cells were then grown for an additional 3 days.

### Osteoclast differentiation assay

Using bone marrow-derived cells isolated from the vehicle control and RANKL-treated mice with administration of salubrinal (0, 1, 2, and 5 μM) in 96-well plates, an osteoclast differentiation assay was performed, as described previously [34,39,40]. For one experimental condition, salubrinal was applied on day 0 to day 6 (6 days), while in the other experimental condition, it was applied on day 4 to day 6 (3 days). Culture medium was exchanged once on day 4 during the 6-day experiments. A tartrate resistant acid phosphate (TRACP)-staining kit was used according to the manufacturer’s instructions to fix and stain adherent cells. TRACP-positive multinuclear cells (> 3 nuclei) were identified as osteoclasts, and their numbers were counted [39]. The osteoclast formation assay was performed at least 3 times using cells isolated independently from different cohorts of mice.

### Osteoclast migration assay

Using a transwell assay, migration of osteoclasts was evaluated as described previously with minor modifications [41]. After isolating them from vehicle control and RANKL-treated mice, bone marrow-derived cells (2 × 10^6^/ml) were cultured in M-CSF and RANKL in 6-well plates for 4 days, and then trypsinized in Hank’s balanced salt solution. With and without salubrinal (2 μM), the osteoclast precursor cells (1 × 10^5^ cells/well) were loaded onto the upper chamber of transwells and allowed to migrate to the bottom chamber through an 8-μm polycarbonate filter coated with vitronectin (Takara Bio Inc., Otsu, Shigma, Japan). α-MEM consisting of 1% bovine serum albumin (BSA) and 30 ng/ml of M-CSF was in the bottom chamber. After reacting for 6 h, the osteoclast precursor cells in the lower chamber was stained with crystal violet and counted.

### Osteoclast adhesion assay

Ninety-six well plates were coated with 5 μg/ml vitronectin in α-MEM supplemented with 30 ng/ml M-CSF and were applied with osteoclast precursors (1 × 10^5^ cells/well) in the presence and absence of salubrinal (2 μM), as described previously [41]. Cells were incubated for 30 min, then washed with PBS three times and fixed with 4% paraformaldehyde at room temperature for 10–15 min. After crystal violet staining, the number of cells adherent to α_v_β_3_ integrin was counted.

### Osteoblast differentiation assay

Bone marrow-derived cells were plated at 2 × 10^6^/ml in 6-well plates in osteogenic differentiation medium (MesenCult proliferation kit) supplemented with 10 nM dexamethasone, 50 μg/ml ascorbic acid 2-phosphate, and 10 mM β-glycerophosphate to induce osteogenic differentiation, as described previously [39,41,42]. Cells were cultured for 2 weeks in the presence and absence of salubrinal (0.5 μM), and medium was changed every other day. For alkaline phosphatase (ALP) staining, cells were fixed in citrate-buffered acetone for 30 s, incubated in the alkaline-dye mix for 30 min, and counterstained with Mayer's Hematoxylin for 10 min. Cells were then evaluated microscopically and the intensity of ALP staining was determined.

To evaluate the effects of RANKL administration on multiple developmental stages starting from bone marrow-derived cells to mature osteoclasts, the RANKL-driven alterations in CFU-GM, CFU-M, osteoclast formation, migration, and adhesion were determined as fold-changes of the RANKL-injected mice to the vehicle control mice. Furthermore, to quantify efficacy of salubrinal in various developmental stages in osteoclastogenesis, the degree of suppression was measured with reduction ratios between the samples treated with and without 2 μM salubrinal.

### Expression analysis of NFATc1 in bone marrow-derived cells and RAW264.7 pre-osteoclast cells

For Western blot analysis, RAW264.7 mouse monocyte/macrophage cells (ATCC, Manassas, VA, USA) were grown in α−MEM containing 10% fetal bovine serum and antibiotics (50 units/ml penicillin, and 50 μg/ml streptomycin; Life Technologies, Grand Island, NY, USA). To induce osteoclastogenesis, 20 ng/ml of RANKL was administered. Bone marrow-derived cells or RAW264.7 cells were lysed in a radioimmunoprecipitation assay (RIPA) lysis buffer, containing protease inhibitors (Santa Cruz Biotechd, Santa Cruz, CA, USA) and phosphatase inhibitos (Calbiochem, Billerica, MA, USA). Isolated proteins were fractionated using 10% SDS gels and electro-transferred to Immobilon-P membranes (Millipore, Billerica, MA, USA). Antibodies specific to NFATc1 (Santa Cruz), and β-actin (Sigma) were employed. Protein levels were assayed using a SuperSignal west femto maximum sensitivity substrate (Thermo Scientific). The bands were scanned with Adobe Photoshop CS2 (Adobe Systems, San Jose, CA, USA) and their intensities were quantified using Image J.

In quantitative PCR, total RNA was extracted using an RNeasy Plus mini kit (Qiagen, Germantown, MD, USA) and reverse transcription was conducted with high capacity cDNA reverse transcription kits (Applied Biosystems, Carlsbad, CA, USA). Real-time PCR was performed using Power SYBR green PCR master mix kits (Applied Biosystems). The PCR primers were: NFATc1 (5′-GGT GCT GTC TGG CCA TAA CT-3′; and 5′-GCG GAA AGG TGG TAT CTC AA-3′), tartrate-resistant acid phosphatase (TRACP) (5′- TCC TGG CTC AAA AAG CAG TT -3′; and 5′- ACA TAG CCC ACA CCG TTC TC -3′); and GAPDH (5′-TGC ACC ACC AAC TGC TTA G-3′; and 5′-GGA TGC AGG GAT GAT GTT C-3′), in which GAPDH was used for internal control. Since TRACP is highly expressed in osteoclasts, we used its mRNA expression level as a marker for development of osteoclasts. The relative mRNA abundance for the selected genes with respect to the level of GAPDH mRNA was expressed as a ratio of *S*_treated_/*S*_control_, where *S*_treated_ is the mRNA level for the cells treated with salubrinal, and *S*_treated_ is the mRNA level for control cells.

### Statistical analysis

The data were expressed as mean ± standard error of mean (SEM). Student’s *t*-test was conducted for two-group comparisons. For many-group comparisons, one-way ANOVA was used, followed by a post-hoc test using Fisher’s protected least significant difference. All comparisons were two-tailed, and statistical significance was assumed at *p* < 0.05. The asterisks (*, **, and ***) represent *p* < 0.05, *p* < 0.01, and *p* < 0.001, respectively.

## Results

### Evaluation of BMD and BMC of the OVX mice and RANKL-injected mice

**Figure 1:**
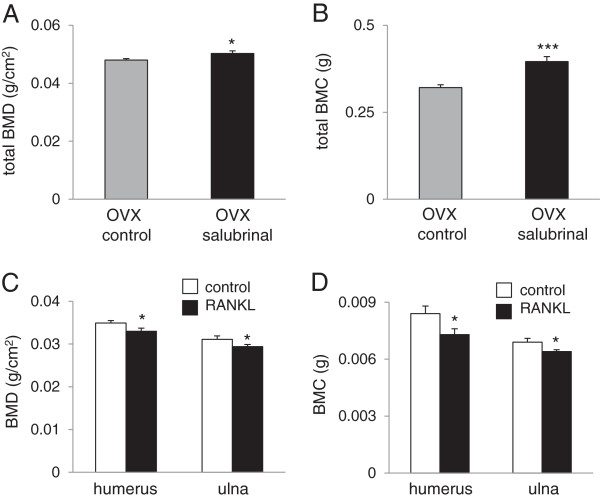
**Determination of BMD and BMC in the OVX mice and RANKL-injected mice. A**: Increase in BMD (g/cm^2^) of the OVX mice by salubrinal (N = 8). **B**: Increase in BMC (g) of the OVX mice by salubrinal (N = 8). **C**: Decrease in BMD (g/cm^2^) of the humerus and ulna of the RANKL-injected mice (N = 6). **D**: Decrease in BMC (g) of the humerus and ulna (N = 6).

Four-week daily administration of salubrinal at a dose of 1 mg/kg to the OVX mice significantly elevated both BMD and BMC of a whole body (Figure 1A-B). Three-day administration of RANKL at a dose of 1 mg/kg, however, significantly decreased BMD and BMC of the humerus and ulna (N = 6; both *p* < 0.05) (Figure 1C-D). Using the RANKL-injected mice, bones from the Iliac, femora, and tibiae were harvested. Bone marrow-derived cells were collected from those bones for examining the effects of salubrinal on developments of osteoclasts and osteoblasts.

### Reduction in the number of CFU-GM by salubrinal in a dosage-dependent manner

**Figure 2:**
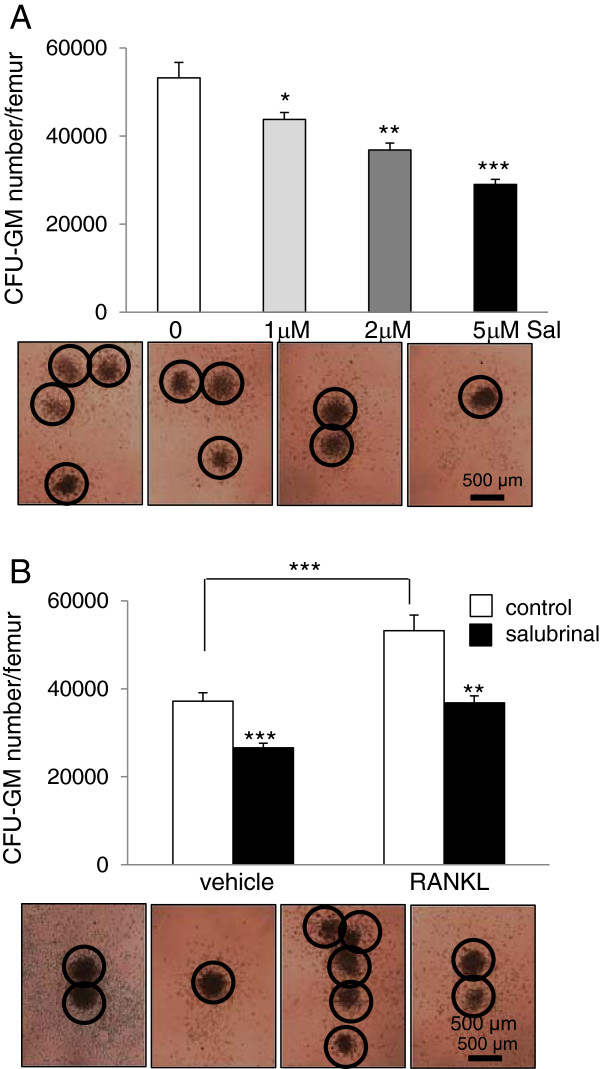
**Effects of salubrinal on colony**-**forming unit-granulocyte**-**macrophage (CFU**-**GM).** Approximately 2.5x10^4^ bone marrow-derived cells were prepared and seeded onto a 35-mm gridded dish supplemented with 30 ng/ml murine M-CSF and 20 ng/ml RANKL. Three dosages of salubrinal (1, 2, and 5 μM) were administered, and cells were cultured for 7 days. **A**: Salubrinal-induced reduction in CFU-GM numbers in the RANKL-injected mice using three dosage of salubrinal. The images exhibit the 4 different CFU-GM cultures, in which the circles indicate the colonies. **B**: Comparison of CFU-GM numbers in the vehicle control and RANKL-injected mice with and without *in vitro* administration of salubrinal. The representative microphotographs are shown, displaying 4 CFU-GM cultures with colonies in circle. Bar = 500 μm.

To determine the effects of salubrinal on the proliferation of osteoclast progenitors, the CFU-GM assay was conducted using bone marrow-derived cells isolated from the RANKL-injected mice. Salubrinal at 1, 2, and 5 μM reduced the total number of CFU-GM in the femur in a dosage-dependent manner (*p* < 0.05 for 1 μM salubrinal; *p* < 0.01 for 2 μM; and *p* < 0.001 for 2 & 5 μM) in the RANKL-injected mice (Figure 2A). The CFU-GM numbers were 37,177 ± 1,919 (vehicle control) and 53,213 ± 3,545 (RANKL administration, *p* < 0.001) (Figure 2B). The CFU-GM numbers were reduced by administration of salubrinal at 2 μM for 7 days by 28.5% (*p* < 0.001) in vehicle control and 30.8% (*p* < 0.001) in the RANKL-injected mice.

### Reduction in the number of CFU-M by salubrinal in a dosage –dependent manner

**Figure 3:**
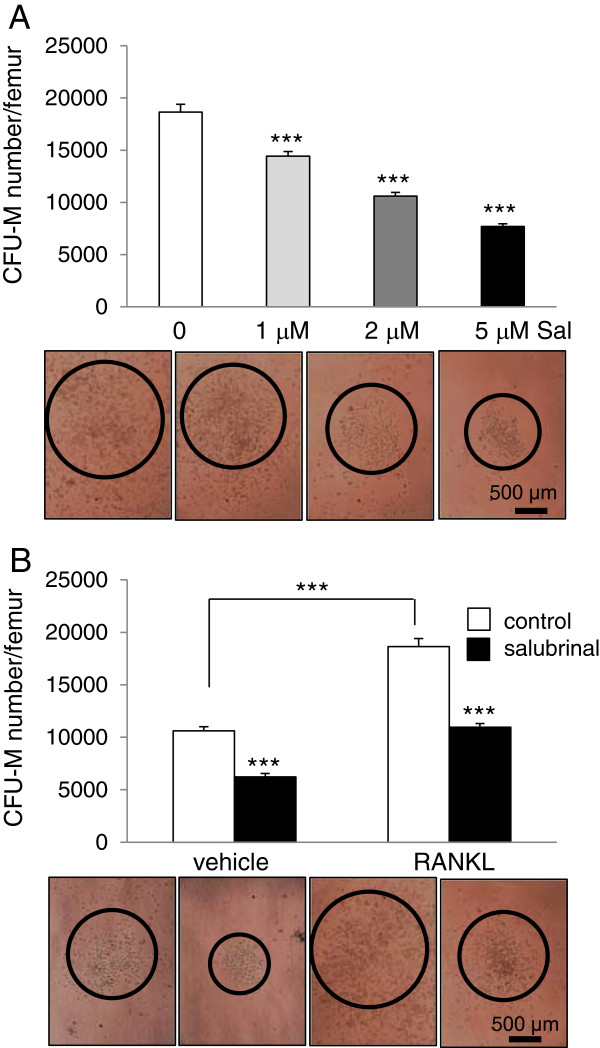
**Effects of salubrinal on colony**-**forming unit**-**macrophage/monocyte (CFU-M).** Approximately 2.5x10^4^ bone marrow mononuclear cells were prepared and seeded onto a 35-mm gridded dish supplemented with 30 ng/ml M-CSF and 20 ng/ml RANKL. Three dosages of salubrinal (1, 2, and 5 μM) were administered and cells were cultured for 7 days. **A**: Salubrinal-induced reduction in CFU-M numbers in the RANKL-injected mice using three dosage of salubrinal. The images exhibit the 4 different CFU-M cultures, in which the circles indicate the colonies. **B**: Comparison of CFU-M numbers in the vehicle control and RANKL-injected mice with and without *in vitro* administration of salubrinal. The representative microphotographs are shown, displaying 4 CFU-M cultures with colonies in circle. Bar = 500 μm.

To determine the effects of salubrinal on the population of osteoclast progenitors, the CFU-M assay was performed using bone marrow-derived cells isolated from the RANKL-injected mice. Consistent with the CFU-M numbers, administration of salubrinal at 1, 2, and 5 μM reduced the total number of CFU-M in the femur in a dosage-dependent manner (all *p* < 0.001 in three dosages) (Figure 3A). The CFU-M numbers were 10,602 ± 396 (vehicle control) and 18,648 ± 760 (RANKL administration, *p* < 0.001) (Figure 3B). Administration of salubrinal at 2 μM for 7 days, for instance, reduced the CFU-M number by 41.2% (*p* < 0.001) in vehicle control and 43.1% (*p* < 0.001) in the RANKL-injected mice.

### Suppression of osteoclast differentiation by salubrinal in a dosage– and time-dependent manner

**Figure 4:**
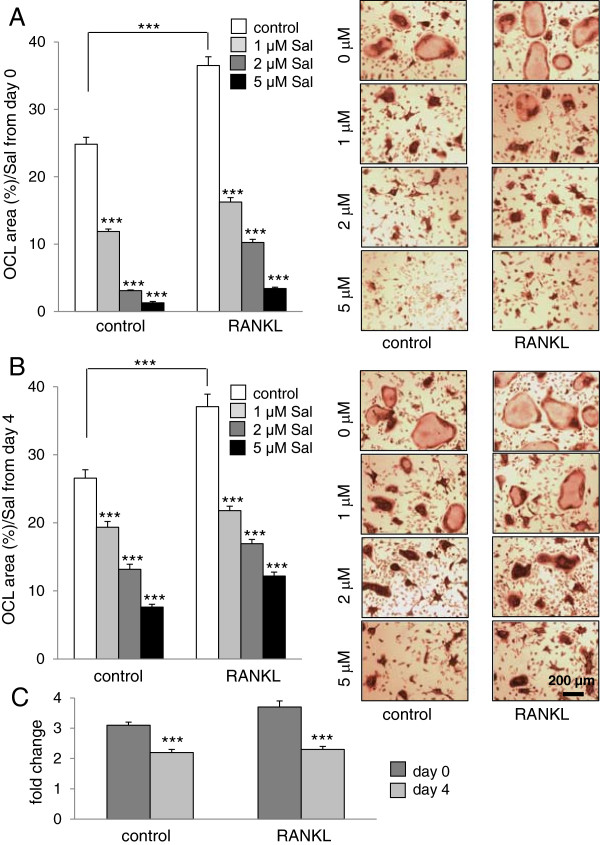
**Effects of administration of salubrinal on multi**-**nucleated osteoclast formation.** Using bone marrow-derived cells isolated from the vehicle control and RANKL-treated mice, an osteoclast differentiation assay was performed. The culture medium was exchanged once on day 4 during the 6-day experiments. TRACP-positive multinuclear cells (> 3 nuclei) were identified as osteoclasts. The areas covered by multi-nucleated osteoclasts are quantified in response to 3 doses of salubrinal (1, 2, and 5 μM). The microphotographs represent the two groups of osteoclast cultures (vehicle control and RANKL administration) with TRACP staining. Bar = 200 μm. **A**: Area covered by multi-nucleated osteoclasts in response to *in vitro* administration of salubrinal from day 0 to day 6 (6 days). **B**: Area covered by multi-nucleated osteoclasts in response to *in vitro* administration of salubrinal from day 4 to day 6 (3 days). **C**: Fold change in response to 2 μM salubrinal.

Compared to the bone marrow-derived cells isolated from the vehicle control, the cells from the RANKL-injected mice exhibited an increase in the surface area occupied by multi-nucleated osteoclasts (24.8 ± 1.0% in vehicle control, and 36.5 ± 1.3% in RANKL administration) (Figure 4A). A series of images show that the process of osteoclast fusion was accelerated by administration of salubrinal. To evaluate the effects of salubrinal, three dosages of salubrinal (1, 2, and 5μM) were applied. In the cultures salubrinal was applied on day 0 for 6 days, administration of salubrinal resulted in a significant decrease in the surface area covered by multi-nucleated osteoclasts for vehicle control (all *p* < 0.001) and RANKL administration (all *p* < 0.001) (Figure 4A). In the cultures salubrinal was applied on day 3 for 4 days, the reduction of the area was also observed (all *p* < 0.001) (Figure 4B). A series of images indicate that the cellular fusion was reduced by salubrinal administration in a time-and dose-dependent manner.

To further evaluate potential effects of the period of salubrinal administration on osteoclast formation, we compared the results of two sets of experiments in which salubrinal at 2 μM was administered from days 0 to 6, and days 4 to 6. The result revealed that salubrinal administration at day 0 presented larger reduction in osteoclast formation than that at day 4 in the vehicle control and RANKL-injected groups (both *p* < 0.001) (Figure 4C).

### Suppression of migration and adhesion of pre-osteoclasts by salubrinal

**Figure 5:**
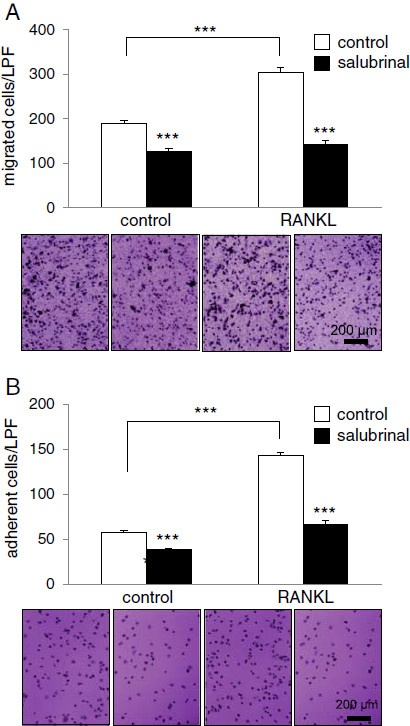
**Effects of salubrinal on migration and adhesion of pre**-**osteoclasts.** Bone marrow-derived cells (2 × 10^6^/ml) were cultured in M-CSF and RANKL in 6-well plates for 4 days to obtain pre-osteoclasts used for the migration and adhesion assays. **A**: Number of migratory cells. Osteoclast precursor cells (1 × 10^5^ cells/well) were loaded onto the upper chamber of transwells in the presence and absence of 2 μM salubrinal. The bottom chamber was filled with α-MEM consisting of 1% BSA and 30 ng/ml of M-CSF, and cells were allowed to migrate to the bottom chamber through an 8-μm polycarbonate filter coated with vitronectin. After reacting for 6 h, the cells in the lower chamber was stained with crystal violet and counted. The images display 2 pairs of osteoclast cultures. Bar = 200 μm. **B**: Number of adherent cells. Ninety-six well plates were coated with 5 μg/ml vitronectin and filled with α-MEM supplemented with 30 ng/ml M-CSF. Approximately 1 × 10^5^ osteoclast precursor cells were cultured per well in the presence and absence of 2 μM salubrinal for 30 min. Cells were stained with crystal violet and the number of cells adherent to α_v_β_3_ integrin was counted. Bar = 200 μm.

Pre-osteoclast cells isolated from the RANKL-injected mice were more migratory (304.1 ± 12.2 cells) than those from the vehicle control (190.4 ± 5.9 cells, *p* < 0.001), and the RANKL-driven increase was 37.4% (Figure 5A). However, salubrinal suppressed the amount of migration by 33.0% in vehicle control (*p* < 0.001) and by 53.2% in RANKL administration (*p* < 0.001). In the M-CSF mediated adhesion assay to α_V_β_3_, the cells isolated from the RANKL-injected mice presented an increase in adhesion by 59.8% (142.5 ± 3.9 cells) over those from the vehicle control (57.3 ± 1.8 cells, *p* < 0.001) (Figure 5B). Administration of salubrinal presented significant reduction in cell adhesion by 32.4% in vehicle control (*p* < 0.001) and by 53.7% in RANKL administration (*p* < 0.001).

### Promotion of osteoblast differentiation by salubrinal

**Figure 6:**
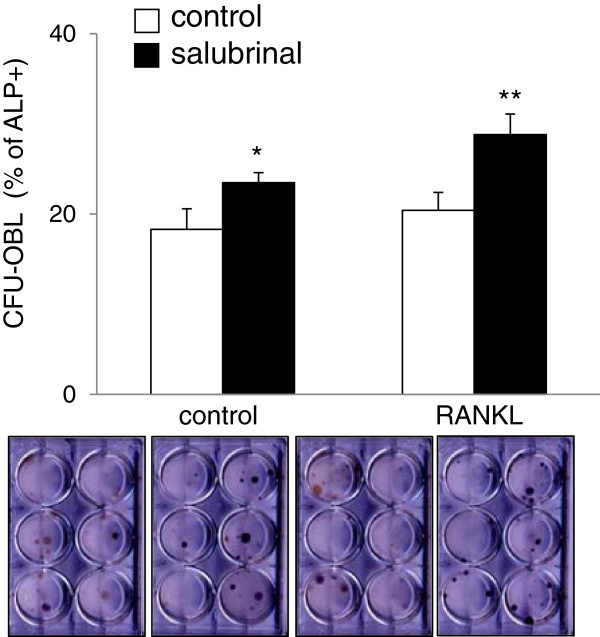
**Enhanced osteoblast development by salubrinal in the CFU**-**OBL assay.** Bone marrow-derived cells were plated at 2 × 10^6^/ml in 6-well plates in osteogenic differentiation medium supplemented with 10 nM dexamethasone, 50 μg/ml ascorbic acid 2-phosphate, and 10 mM β-glycerophosphate. Cells were cultured for 2 weeks in the presence and absence of 0.5 μM salubrinal, and medium was changed every other day. For alkaline phosphatase (ALP) staining, cells were fixed in citrate-buffered acetone for 30 s, incubated in the alkaline-dye mix for 30 min, and counterstained with Mayer’s Hematoxylin for 10 min. Cells were then evaluated microscopically and the intensity of ALP staining was determined. The images display 2 pairs of osteoblast cultures.

In the CFU-OBL assay, a significant increase in the number of ALP positive cells was detected by administration of salubrinal. Without salubrinal, the percentage of ALP-positive cells was 18.3 ± 2.3% in vehicle control and 20.4 ± 2.0% in RANKL administration (*p* < 0.001) (Figure 6). Administration of salubrinal at 0.5 μM increased the percentage of ALP-positive cells to 23.5 ± 1.1% in vehicle control (*p* < 0.05) and 28.8 ± 2.3% in RANKL administration (*p* < 0.01) (Figure 6).

### Downregulation of NFATc1 by salubrinal in bone marrow-derived cells and RAW264.7 pre-osteoclast cells

**Figure 7:**
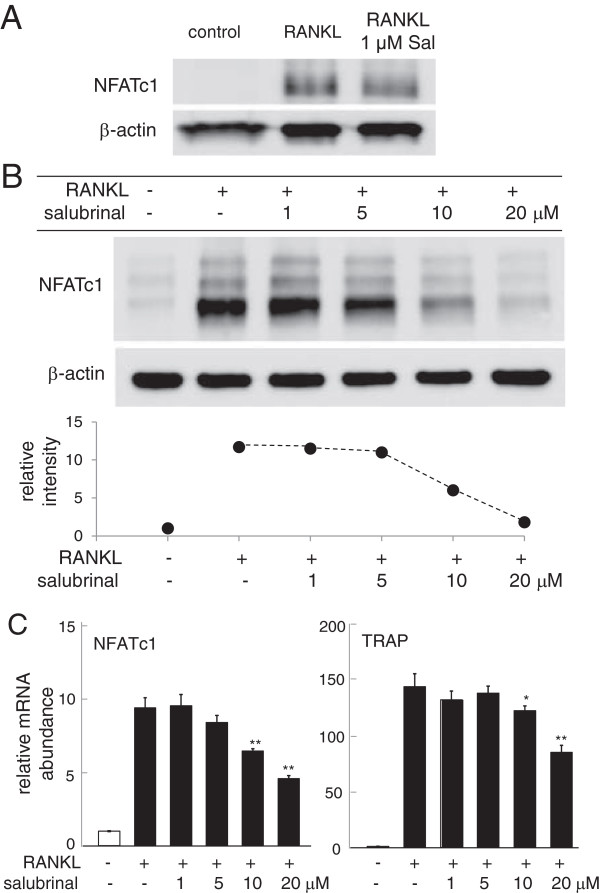
**Suppression of RANKL-induced expression of NFATc1 and TRACP by salubrinal in RAW264.7 cells. A**: Salubrinal-driven suppression of NFATc1 expression in RANKL-stimulated bone marrow-derived cells. **B**: Dose dependent suppression of NFATc1 in response to 1 – 20 μM salubrinal. β-actin was used as a loading control. **C**: Relative mRNA levels of NFATc1 and TRACP in response to 1 – 20 μM salubrinal. The mRNA levels are normalized by the mRNA level of the sample without salubrinal in the absence of RANKL stimulation.

Bone marrow-derived cells were incubated with RANKL in the presence and absence of salubrinal. Incubation with 20 ng/ml RANKL markedly increased the level of NFATc1, a master transcription factor for development of osteoclasts, and administration of 1 μM salubrinal reduced the RANKL-driven increase in NFATc1 by 24% (Figure 7A). To further evaluate the effects of salubrinal, we employed RAW264.7 pre-osteoclast cells. Administration of 20 ng/ml RANKL elevated the level of NFATc1, and in response to 1–20 μM salubrinal the RANKL-induced elevation of NFATc1 was reduced in a dose dependent fashion (Figure 7B). Furthermore, the mRNA levels of NFATc1 and TRACP were increased by RANKL, and their elevation was suppressed by administration of salubrinal (Figure 7C).

## Discussion

The present study presents the beneficial effect of *in vivo* administration of salubrinal on BMD and BMC of the OVX mice, and *in vitro* effects on the culture of bone marrow-derived cells isolated from the RANKL-injected and control mice. In the osteoclast assays of CFU-GM, CFU-M, and formation of multi-nucleation, salubrinal significantly reduced the numbers of osteoclastic colonies and cells isolated from both the vehicle control and RANKL-injected mice. In the two sets of maturation assays, in which salubrinal was applied from day 0 to 6 and from day 4 to 6, it suppressed both the early and late stages of osteoclastogenesis. This suppressive effect was larger in the cells isolated from the RANKL-injected mice than the vehicle control mice. In addition to attenuating osteoclastogenesis, salubrinal was able to reduce adhesion and migration of osteoclasts. Furthermore, it increased the number of CFU-OBL colonies suggesting that it not only inhibits development of osteoclasts but also promotes development of osteoblasts. Quantitative PCR and Western blot analysis revealed that the mRNA and protein levels of NFATc1 were elevated by RANKL, and this elevation was suppressed by administration of salubrinal in a dose dependent fashion.

In evaluating the effects of salubrinal on fates of HSCs and MSCs in bone marrow-derived cells, we employed the recently developed RANKL administration model of osteoporosis. An advantage of this RANKL administration model includes a short period (3 days in this study) for induction of osteoclastogenesis, and activation of multiple steps in the development of osteoclasts. In the RANK/RANKL/OPG signaling pathway, RANKL regulates not only development of osteoclasts but also their activation and survival [43]. RANKL is expressed in bone, bone marrow, and lymphoid tissues including spleen that houses osteoclast precursor cells as macrophages [44]. The RANKL administration model provided a platform to evaluate efficacy of salubrinal as a potential therapeutic agent for preventing osteoclastogenesis and bone resorption. Besides bone resorption, however, RANKL is involved in multiple functions in the immune system such as proliferation of T cells and inhibition of apoptosis of dendritic cells [45]. It is reported that overproduction of RANKL induces inflammatory bone disorders [46,47]. Thus, the results from any animal model including the RANKL administration model should be confirmed by other animal models and eventually clinical trial.

The regulatory mechanism of salubrinal’s action on osteoclastogenesis is not well understood. Salubrinal is known as an inhibitor of serine/threonine-protein phosphatase PP1 and it elevates the phosphorylation level of eIF2α (eIF2α-p) [48]. The level of eIF2α-p is upregulated in response to various stresses including viral infection, nutrient deprivation, radiation, and stress to the endoplasmic reticulum [49]. To cope with these cellular insults and reduce apoptosis, the elevated eIF2α-p in general lowers ribosome’s efficiency of protein synthesis except for a group of proteins such as ATF4. Applications of salubrinal have been reported to reduce stress induced apoptosis [50]. We have previously shown that partial silencing of eIF2α by RNA interference reduces salubrinal-driven downregulation of NFATc1 in RAW264.7 cells [51], and the results in this study indicate that mRNA and protein expression of NFATc1 is downregulated by salubrinal. NFATc1 is a member of the NFAT transcription factor family and a master transcription factor for osteoclast development. It is reported that NFATc1-deficient embryonic stem cells are unable to differentiate into osteoclasts [52]. He et al. has recently shown that NFATc1 expression is regulated at a translational stage in bone marrow macrophage cells, and a phosphorylation mutant plasmid for eIF2α restored RANKL-induced NFATc1 expression [53]. MafB (V-maf musculoaponeurotic fibrosarcoma oncogene homolog B), IRF8 (interferon regulatory factor 8), and Bcl6 (V cell lymphoma) have been mentioned as inhibitors of NFATc1 [54-56]. Further analysis is necessary for identification of the mechanism of salubrinal’s action on NFATc1, which is possibly regulated by eIF2α alone or any other mediators.

## Conclusions

It is premature to draw any conclusion on development of a potential therapeutic agent for treatment of osteoporosis, but salubrinal possesses several unique features. First, it is a small synthetic chemical agent, which can be taken as an oral pill. Second, it has a dual role of stimulation of bone formation and attenuation of bone resorption. Third, its effects are stronger in the cells isolated from the osteoporotic RANKL-injected mice than those from the control mice. Fourth, it presents dose dependent efficacy in preventing osteoclastogenesis throughout a developmental stage including proliferation, multi-nucleation, and maturation, as well as migration and adhesion. The results herein support the possibility of preventing bone loss through salubrinal-driven regulation of bone marrow-derived cells.

## Abbreviations

OVX: Ovariectomy; BMD: Bone mineral density; BMC: Bone mineral content; eIF2α: Eukaryotic translation initiation factor 2 alpha; eIF2α-p: Phosphorylated eIF2α; NFκB: Nuclear factor kappa B; ATF4: Activating transcription factor 4; NFATc1: Nuclear factor of activated T cells c1; OPG: Osteoprotegerin; RANKL: A Receptor activator of nuclear factor kappa-B ligand; M-CSF: Murine macrophage-colony stimulating factor; BMMNCs: Bone marrow mononuclear cells; MSCs: Mesenchymal stem cells; CFU-M: Colony forming-unit macrophages; CFU-GM: Colony forming-unit granulocyte/macrophages; CFU-OBL: Colony-forming unit – osteoblasts; ALP: Alkaline phosphatase; TRACP: Tartrate resistant acid phosphate; BSA: Bovine serum albumin.

## Competing interests

The authors declare that they have no competing interests.

## Authors’ contributions

HY participated in experimental designs, and drafted a manuscript. KH conducted molecular experiments, performed data collection and analysis. AC and TD conducted animal experiments. NT and AA assisted data collection. PZ participated in experimental designs, performed animal and cell experiments, conducted data collection and interpretation, and drafted a manuscript. PZ accepted responsibility for integrity of data analysis. All authors read and approved the final manuscript.

## Pre-publication history

The pre-publication history for this paper can be accessed here:

http://www.biomedcentral.com/1471-2474/14/197/prepub

## References

[B1] van den Bergh JP van Geel TA Geusens PP Osteoporosis, frailty and fracture: implications for case finding and therapy Nat Rev Rheumatol 2012 8 163 172 10.1038/nrrheum.2011.217 22249162

[B2] Shiraki M Kuroda T Miyakawa N Fujinawa N Tanzawa K Ishizuka A Tanaka S Tanaka Y Hosoi T Itoi E Morimoto S Itabashi A Sugimoto T Yamashita T Gorai I Mori S Kishimoto H Mizunuma H Endo N Nishizawa Y Takaoka K Ohashi Y Ohta H Fukunaga M Nakamura T Orimo H Design of a pragmatic approach to evaluate the effectiveness of concurrent treatment for the prevention of osteoporotic fractures: rationale, aims and organization of a Japanese Osteoporosis Intervention Trial (JOINT) initiated by the Research Group of Adequate Treatment of Osteoporosis (A-TOP) J Bone Miner Metab 2011 29 37 43 10.1007/s00774-010-0188-x 20461422

[B3] Vercini F Grimaldi F PTH 1–84: bone rebuilding as a target for the therapy of severe osteoporosis Clin Cases Miner Bone Metab 2012 9 31 36 22783333 PMC3392676

[B4] Dempster DW Lambing CL Kostenuik PJ Grauer A Role of RANK ligand and denosumab, a targeted RANK ligand inhibitor, in bone health and osteoporosis: a review of preclinical and clinical data Clin Ther 2012 34 521 536 10.1016/j.clinthera.2012.02.002 22440513

[B5] Yang Q Jian J Abramson SB Huang X Inhibitory effects of iron on bone morphogenetic protein 2-induced osteoblastogenesis J Bone Miner Res 2011 26 1188 1196 10.1002/jbmr.337 21308772

[B6] Khosla S Bilezikian JP Dempster DW Lewiecki EM Miler PD Neer RM Recker RR Shane E Shoback D Potts JT Benefits and risks of bisphosphonate therapy for osteoporosis J Clin Endocrinol Metab 2012 97 2272 2282 10.1210/jc.2012-1027 22523337

[B7] Riggs BL Khosla S Melton LJ III Sex steroids and the construction and conservation of the adult skeleton Endocr Rev 2002 23 279 302 10.1210/er.23.3.279 12050121

[B8] Valverde P Pharmacotherapies to manage bone loss-associated diseases: a quest for the perfect benefit-to-risk ratio Curr Med Chem 2008 15 284 304 10.2174/092986708783497274 18288984

[B9] Boras-Granic K Wysolmerski JJ PTHrP and breast cancer: more than hypercalcemia and bone metastases Breast Cancer Res 2012 14 307 10.1186/bcr3129 22546075 PMC3446368

[B10] Boyce M Bryant KF Jousse C Long K Harding HP Scheuner D Kaufman RJ Ma D Coen DM Ron D Yuan J A selective inhibitor of eIF2alpha dephosphorylation protects cells from ER stress Science 2005 307 935 939 10.1126/science.1101902 15705855

[B11] Huang X Chen Y Zhang H Ma Q Zhang YW Xu H Salubrinal attenuates β-amyloid-induced neuronal death and microglial activation by inhibition of the NF-κB pathway Neurobiol Aging 2012 33 1007.e9 1007.e17 10.1016/j.neurobiolaging.2011.10.007 22056200 PMC3294262

[B12] Saito A Ochiai K Kondo S Tsumagari K Murakami T Cavener DR Imaizumi K Endoplasmic reticulum stress response mediated by the PERK-eIF2(alpha)-ATF4 pathway is involved in osteoblast differentiation induced by BMP2 J Biol Chem 2011 286 4809 4818 10.1074/jbc.M110.152900 21135100 PMC3039352

[B13] Zhang P Hamamura K Jiang C Zhang L Yokota H Salubrinal promotes healing of surgical wounds in rat femurs J Bone Miner Metab 2012 30 568 579 10.1007/s00774-012-0359-z 22610062

[B14] Li X Ling W Khan S Yaccoby S Therapeutic effects of intrabone and systemic mesenchymal stem cell cytotherapy on myeloma bone disease and tumor growth J Bone Miner Res 2012 27 1635 1648 10.1002/jbmr.1620 22460389 PMC3395777

[B15] Sun L Peng Y Sharrow AC Iqbal J Zhang Z Papachristou DJ Zaidi S Zhu LL Yaroslavskiy BB Zhou H Zallone A Sairam MR Kumar TR Bo W Braun J Cardoso-Landa L Schaffler MB Moonga BS Blair HC Zaidi M FSH directly regulates bone mass Cell 2006 125 247 260 10.1016/j.cell.2006.01.051 16630814

[B16] Weitzmann MN Pacifici R Estrogen deficiency and bone loss: an inflammatory tale J Clin Invest 2006 116 1186 1194 10.1172/JCI28550 16670759 PMC1451218

[B17] Shahnazari M Wronski T Chu V Williams A Leeper A Stolina M Ke HZ Halloran B Early response of bone marrow osteoprogenitors to skeletal unloading and sclerostin antibody Calcif Tissue Int 2012 91 50 58 10.1007/s00223-012-9610-9 22644321

[B18] Zhang P Hammamura K Yokota H A brief review of bone adaptation to unloading Genomics Proteomics Bioinf 2008 6 4 7 10.1016/S1672-0229(08)60016-9 PMC5054086 18558381

[B19] Gaspar AP Lazaretti-Castro M Brandão CM Bone mineral density in spinal cord injury: an evaluation of the distal femur J Osteoporos 2012 2012 519754 22970408 10.1155/2012/519754 PMC3434402

[B20] Jiang SD Jiang LS Dai LY Mechanisms of osteoporosis in spinal cord injury Clin Endocrinol (Oxf) 2006 65 555 565 10.1111/j.1365-2265.2006.02683.x 17054455

[B21] Omi N Ezawa I Animal models for bone and joint disease. Low calcium diet-induced rat model of osteoporosis Clin Calcium 2012 21 173 180 21289413

[B22] Chennaiah S Vijayalakshmi V Suresh C Effect of the supplementation of dietary rich phytoestrogens in altering the vitamin D levels in diet induced osteoporotic rat model J Steroid Biochem Mol Biol 2010 121 268 272 10.1016/j.jsbmb.2010.03.070 20362669

[B23] Lo Iacono N Blair HC Poliani PL Marrella V Ficara F Cassani B Facchetti F Fontana E Guerrini MM Traggiai E Schena F Paulis M Mantero S Inforzato A Valaperta S Pangrazio A Crisafulli L Maina V Kostenuik P Vezzoni P Villa A Sobacchi C Osteopetrosis rescue upon RANKL administration to Rankl(−/−) mice: A new therapy for human RANKL-dependent ARO J Bone Miner Res 2012 27 2501 2510 10.1002/jbmr.1712 22836362

[B24] Campbell GM Ominsky MS Boyd SK Bone quality is partially recovered after the discontinuation of RANKL administration in rats by increased bone mass on existing trabeculae: an in vivo micro-CT study Osteoporosis Int 2011 22 931 942 10.1007/s00198-010-1283-5 20480144

[B25] Yasuda H Animal models for bone and joint disease. RANKL-injected bone loss model Clin Calcium 2011 21 197 208 21289416

[B26] Tomimori Y Mori K Koide M Nakamichi Y Ninomiya T Udagawa N Yasuda H Evaluation of pharmaceuticals with a novel 50-hour animal model of bone loss J Bone Miner Res 2009 24 1194 1205 10.1359/jbmr.090217 19257825

[B27] Wasilewska A Rybi-Szuminska AA Zoch-Zwierz W Serum osteoprotegrin (OPG) and receptor activator of nuclear factor kappaB (RANKL) in healthy children and adolescents J Pediatr Endocrinol Metab 2009 22 1099 1104 20333868 10.1515/jpem.2009.22.12.1099

[B28] Takayanagi H Osteoimmunology and the effects of the immune system on bone Nat Rev Rheumatol 2009 5 667 676 10.1038/nrrheum.2009.217 19884898

[B29] Nakamura M Udagawa N Osteoporosis and RANKL signal Clin Calcium 2011 21 1149 1155 21814019

[B30] Perlot T Penninger JM Development and function of murine B cells lacking RANK J Immunol 2012 188 1201 1205 10.4049/jimmunol.1102063 22219325

[B31] Mun SH Won HY Hernandez P Aguila HL Lee SK Deletion of CD74, a putative MIF receptor, in mice enhances osteoclastogenesis and decreases bone mass J Bone Miner Res 2013 28 948 959 10.1002/jbmr.1787 23044992 PMC3563845

[B32] Droxmeyer HE Kappes F Mor-Vaknin N Legendre M Kinzforql J Cooper S Hangoc G Markovitz DM DEK regulates hematopoietic stem engraftment and progenitor cell proliferation Stem Cells Dev 2012 21 1449 1454 10.1089/scd.2011.0451 21943234 PMC3359622

[B33] Kroepfl JM Pekovits K Stelzer I Fuchs R Zelzer S Hofmann P Sedlmayr P Dohr G Wallner-Liebmann S Domej W Muller W Exercise increases the frequency of circulating hematopoietic progenitor cells, but reduces hematopoietic colony-forming capacity Stem Cells Dev 2012 21 2915 2925 10.1089/scd.2012.0017 22616638

[B34] He Y Rhodes SD Chen S Wu X Yuan J Yang X Jiang L Li X Takahashi N Xu M Mohammad KS Guise TA Yang FC c-Fms Signaling Mediates Neurofibromatosis Type-1 Osteoclast Gain-In-Functions PLoS One 2012 7 e46900 10.1371/journal.pone.0046900 23144792 PMC3492362

[B35] Yan D Gurumurthy A Wright M Pfeiler TW Loboa EG Everett ET Genetic background influences fluoride’s effects on osteoclastogenesis Bone 2007 41 1036 1144 10.1016/j.bone.2007.07.018 17936699 PMC2238641

[B36] Broxmeyer HE Mejia JA Hangoc G Barese C Dinauer M Cooper S SDF-1/CXCL12 enhances in vitro replating capacity of murine and human multipotential and macrophage progenitor cells Stem Cells Dev 2007 16 589 596 10.1089/scd.2007.0044 17784832

[B37] McHugh KP Shen Z Crotti TN Flannery MR Fajardo R Bierbaum BE Goldring SR Role of cell-matrix interactions in osteoclast differentiation Adv Exp Med Biol 2007 602 107 111 10.1007/978-0-387-72009-8_14 17966395

[B38] Wu X Chen S Orlando SA Yuan J Kim ET Munugalavadla V Mali RS Kapur R Yang FC p85α regulates osteoblast differentiation by cross-talking with the MAPK pathway J Biol Chem 2011 286 13512 13521 10.1074/jbc.M110.187351 21324896 PMC3075697

[B39] Abdallah BM Ditzel N Mahmood A Isa A Traustadottir GA Schilling AF Ruiz-Hidalgo MJ Laborda J Amling M Kassem M DLK1 is a novel regulator of bone mass that mediates estrogen deficiency-induced bone loss in mice J Bone Miner Res 2011 26 1457 1471 10.1002/jbmr.346 21308776

[B40] Jansen ID Vermeer JA Bloemen V Stap J Everts V Osteoclast fusion and fission Calcif Tissue Int 2012 90 515 522 10.1007/s00223-012-9600-y 22527205 PMC3349023

[B41] Xiao G Cheng H Cao H Chen K Tu Y Yu S Jiao H Yang S Im HJ Chen D Chen J Wu C Critical role of filamin-binding LIM protein 1 (FBLP-1)/migfilin in regulation of bone remodeling J Biol Chem 2012 287 21450 21460 10.1074/jbc.M111.331249 22556421 PMC3375566

[B42] Nabavi N Khandani A Camirand A Harrison RE Effects of microgravity on osteoclast bone resorption and osteoblast cytoskeletal organization and adhesion Bone 2011 49 965 974 10.1016/j.bone.2011.07.036 21839189

[B43] Boyce BF Xing L The RANKL/RANK/OPG pathway Curr Osteoporos Rep 2007 5 98 104 10.1007/s11914-007-0024-y 17925190

[B44] Graham LS Tintut Y Parhami F Kitchen CM Ivanov Y Tetradis S Effros RB Bone density and hyperlipidemia: the T-lymphocyte connection J Bone Miner Res 2010 25 2460 2469 10.1002/jbmr.148 20533376 PMC3179287

[B45] Akiyama T Shinzawa M Akiyama N RANKL-RANK interaction in immune regulatory systems World J Orthop 2012 3 142 150 10.5312/wjo.v3.i9.142 23173110 PMC3502610

[B46] Chang SK Noss EH Chen M Gu Z Townsend K Grenha R Leon L Lee SY Lee DM Brenner MB Cadherin-11 regulates fibroblast inflammation Proc Natl Acad Sci U S A 2011 108 8402 8407 10.1073/pnas.1019437108 21536877 PMC3100978

[B47] Belibasakis GN Reddi D Bostanci N Porphyromonas gingivalis induces RANKL in T-cells Inflammation 2011 34 133 138 10.1007/s10753-010-9216-1 20446027

[B48] Vander Mierde D Scheuner D Quintens R Patel R Song B Tsukamoto K Beullens M Kaufman RJ Bollen M Schuit FC Glucose activates a protein phosphatase-1-mediated signaling pathway to enhance overall translation in pancreatic beta-cells Endocrinology 2007 148 609 617 17082262 10.1210/en.2006-1012

[B49] Dey S Baird TD Zhou D Palam LR Spandau DF Wek RC Both transcriptional regulation and translational control of ATF4 are central to the integrated stress response J Biol Chem 2010 285 33165 33174 10.1074/jbc.M110.167213 20732869 PMC2963398

[B50] Dou G Sreekumar PG Spee C He S Ryan SJ Kannan R Hinton DR Deficiency of αB crystallin augments ER stress-induced apoptosis by enhancing mitochondrial dysfunction Free Radic Biol Med 2012 53 1111 1122 10.1016/j.freeradbiomed.2012.06.042 22781655 PMC3454510

[B51] Hamamura K Tanjung N Yokota H Suppression of osteoclastogenesis through phosphorylation of eukaryotic translation initiation factor 2 alpha J Bone Miner Metab 2013 in press 10.1007/s00774-013-0450-0 23536193

[B52] Takayanagi H The role of NFAT in osteoclast formation Ann NY Acad Sci 2007 1116 227 237 10.1196/annals.1402.071 18083930

[B53] He L Lee J Jang JH Sakchaisri K Hwang J Cha-Molstad HJ Kim KA Ryoo IJ Lee HG Kim SO Soung NK Lee KS Kwon YT Erikson RL Ahn JS Kim BY Osteoporosis regulation by salubrinal through eIF2**α** mediated differentiation of osteoclast and osteoblast Cell Signal 2013 25 552 560 10.1016/j.cellsig.2012.11.015 23178987 PMC3593652

[B54] Kim K Kim JH Lee J Jin HM Kook H Kim KK Lee SY Kim N MafB negatively regulates RANKL-mediated osteoclast differentiation Blood 2007 109 3253 3259 10.1182/blood-2006-09-048249 17158225

[B55] Zhao B Takami M Yamada A Wang X Koga T Hu X Tamura T Ozato K Choi Y Ivashkiv LB Takayanagi H Kamijo R Interferon regulatory factor-8 regulates bone metabolism by suppressing osteoclastogenesis Nat Med 2009 15 1066 1071 10.1038/nm.2007 19718038 PMC2755267

[B56] Miyauchi Y Ninomiya K Miyamoto H Sakamoto A Iwasaki R Hoshi H Miyamoto K Hao W Yoshida S Morioka H Chiba K Kato S Tokuhisa T Saitou M Toyama Y Suda T Miyamoto T The Blimp1-Bcl6 axis is critical to regulate osteoclast differentiation and bone homeostasis J Exp Med 2010 207 751 762 10.1084/jem.20091957 20368579 PMC2856022

